# Effect of wearing diapers on toddler’s gait

**DOI:** 10.1038/s41598-021-99583-4

**Published:** 2021-10-11

**Authors:** Tomoya Ueda, Haruna Asano, Kyoko Tsuge, Kanako Seo, Motoki Sudo, Yuko Fukuda, Yasuyuki Okuda, Kiyoshi Kataoka, Hiroyuki Iwasaki, Hisashi Naito, Da Jiang Lu

**Affiliations:** 1 Tokyo Research Laboratories, Kao Corporation, 2-1-3 Bunka, Sumida-ku, Tokyo, 131-8501 Japan; 2 Tochigi Research Laboratories, Kao Corporation, 2606 Akabane, Ichikai-machi, Haga-gun, Tochigi, 321-3497 Japan; 3 Jujo Pediatric Clinic, 3-22-8-101 Kamijujo, Kita-ku, Tokyo, 114-0034 Japan; 4 Graduate School of Health and Sports Science, Juntendo University, 1-1 Hirakagakuendai, Inzai, Chiba 270-1695 Japan; 5 Department of Human Sports Science, Shanghai University of Sport, Shanghai, 200438 China

**Keywords:** Health care, Medical research

## Abstract

Gait maturation in infants develops gradually through several phases. However, external factors such as childrearing practices, especially the wearing of diapers, may affect an infant’s motor development. This study investigated the influence of different bulk stresses on the gait of toddlers wearing a disposable diaper. Twenty-six healthy toddlers (age: 19.2 ± 0.9 months) participated in this study. We measured the joint kinematics (pelvis angle and hip-joint angle) and spatiotemporal parameters (step length and step width) of the toddlers’ gait under four dress conditions (wearing Type A_WET, Type A_DRY, and Type B_WET diapers and naked). Type B_WET had a higher bulk stress than Type A_WET, and Type A_DRY had lower stress than Type A _ WET. Our results indicate that the walk of toddlers when wearing a diaper differs from that when naked. This difference is due to the effect of the bulk of the diaper on the lower limb. A high bulk stress has a greater influence than that of a low bulk stress on joint dynamics and step width. Therefore, our findings suggest that wearing diapers with high bulk stress may inhibit the natural gait patterns of toddlers.

## Introduction

Independent mobility is an essential requirement for executing various motor functions in infancy, which are associated with an early stage of gait pattern development. Gait is a representative parameter in normal motor development^1^. Since 1960, gait assessments and analyses of toddlers have been conducted at the onset of independent walking abilities to evaluate the development of different locomotion patterns and coordination. Such analyses have proven useful in terms of influencing the decision-making process in the case of interventions required, and for the strategic formulation of treatment plans^2–10^. During the first few years of independent walking, considerable changes occur in gait parameters, specifically in terms of spatiotemporal parameters, joint kinematics, and dynamics^10–14^. Therefore, it is important to account for these changes and modifications to understand the development of mature gait patterns during the development stage in toddlers^8,15^.

Gait maturation begins at approximately 12 months of age, and gradual development toward mature walking occurs through a series of phases in the first three years of life: newborn stepping, infant supported walking, and infant independent walking^16^. However, during these early stages of gait development, external factors such as childrearing practices—specifically the use of diapers—may affect an infant’s motor development. Previous studies have reported that wearing a disposable diaper restrains an infant’s movements and mobility, thereby reducing the amount of their physical activity and restricting motor development. Gima et al. demonstrated that the shape of the diaper affects the spontaneous movements of the lower limbs in young infants^17^. Additionally, Cole et al. concluded that wearing diapers may decrease the gait proficiency in toddlers aged between 13 and 19 months^18^. This is because infants that wear diapers can only take wider and smaller steps, and thus achieve smaller dynamic base angles. All these conditions are indicative of unstable gait patterns, as compared to those when walking naked—that is, without diapers. Therefore, the shape and bulk of disposable diapers are considered to have significant effects on the movement of the lower limbs in infants.

However, this condition has not been sufficiently considered, and a wide variety of analyses is needed for clarification. To verify the prominent effects of diapers on a growing infant, although joint kinematics and dynamics in gait may be important information, prior research conducted using spatiotemporal parameters and trajectory data exclusively has not reported on the joint kinematics and dynamics of the lower limbs. Furthermore, we have to consider various diaper conditions. Diapers in previous studies were employed only in the dry condition (prior to urination). However, many parents rarely change the disposable diaper immediately if the infant has only urinated. The shape and bulk of diapers in the wet condition differ from those in the dry condition. In particular, the stress of the bulk on the lower limbs is dependent on the condition of the diapers. These factors necessitate clarification regarding the effects of wearing diapers on the joint kinematics and dynamics of gait in toddlers. They also prompt the question of whether different bulk stress characteristics affect the movement of a toddler’s lower limbs.

Therefore, the present study aims to investigate the influence of different levels of bulk stress on the gait of toddlers that wear a disposable diaper. We hypothesized that a toddlers’ gait while wearing a diaper differs from that while walking naked, owing to the diaper’s effects on the lower limbs; for example, a situation where the resulting high bulk stress has a greater effect on a toddlers’ gait than low bulk stress. Clarifying the bulk stress on toddlers’ gait will help in optimizing the designs of diapers so as to improve the gait and motor development of toddlers.

## Results

Table 1 presents the specifications of the diapers (Types A and B) used in the present study. We chose two types of diapers with different compressive loads for bulk, which are adjusted for the rate of increase of the bulk and easy bending of the bulk^25^.

**Table 1:** Specifications of each diaper.

	Type A_DRY	Type A_WET	Type B_WET
Waist belt (mm)	120	120	110
Crotch belt (mm)	125	125	95
Compressive load for bulk (N)	0.82	7.85	11.46

### Pelvis angle

**Figure 1:**
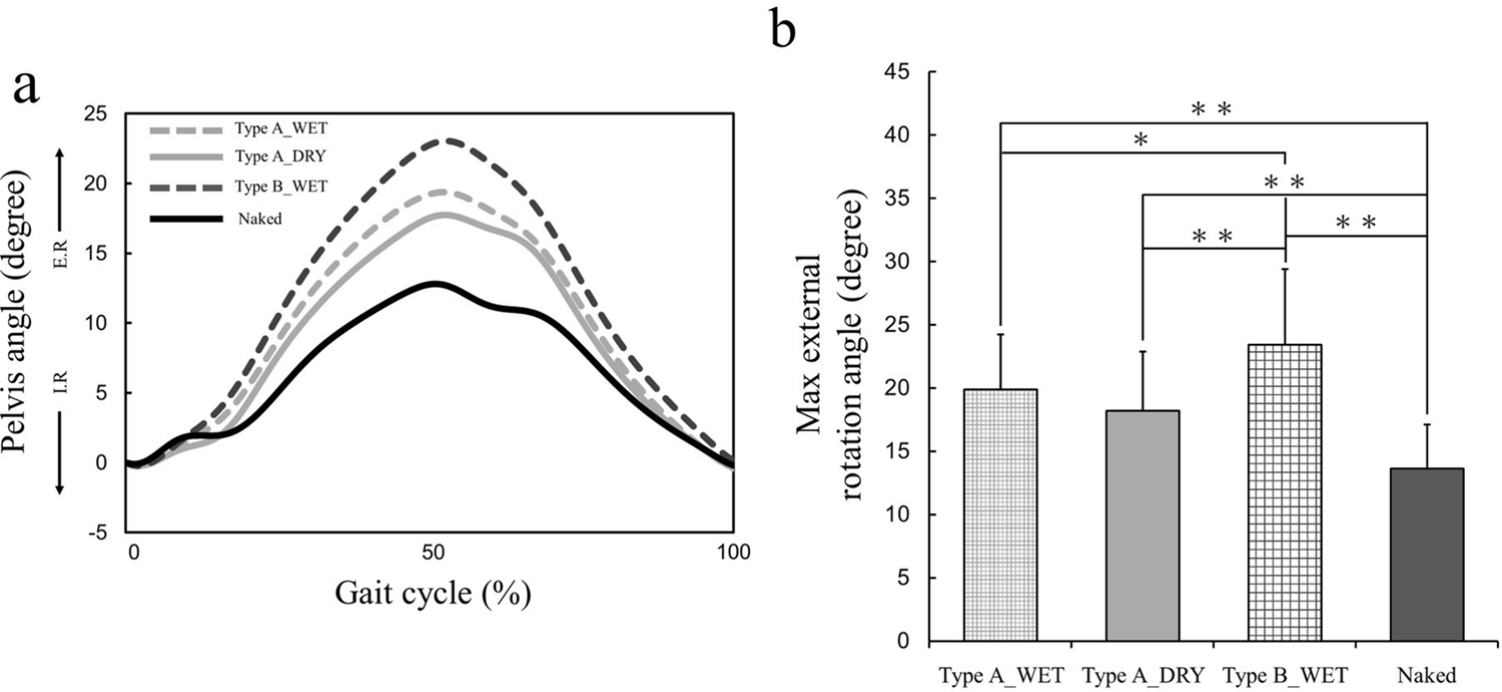
(**a**) Mean time series across subjects at each condition of the pelvic angle in the horizontal plane during a gait cycle. *I.R.* internal rotation, *E.R.* external rotation. (**b**) Mean and standard deviation of the mean of the normalized maximum external rotation angle on the pelvis for the four dress conditions. *p < 0.05; **p < 0.01.

Figure 1a shows a plot of the average pelvis angle in the horizontal plane (external/internal rotation) in a gait cycle. Wearing diapers under wet conditions results in greater external rotation than wearing diapers under dry conditions and being naked (i.e., not wearing a diaper). Figure 1b shows the maximum external rotation of the pelvis. The statistical analysis revealed significant intercondition differences in the maximum external rotation on the pelvis (*F*_(3, 75)_ = 42.80, *p* < 0.01, ES = 0.63), specifically among dress conditions, except between Type A_WET and Type A_DRY. Wearing Type B_WET had a larger maximum external rotation on the pelvis when compared with the other three conditions (*p* < 0.05).

### Hip joint angle

**Figure 2:**
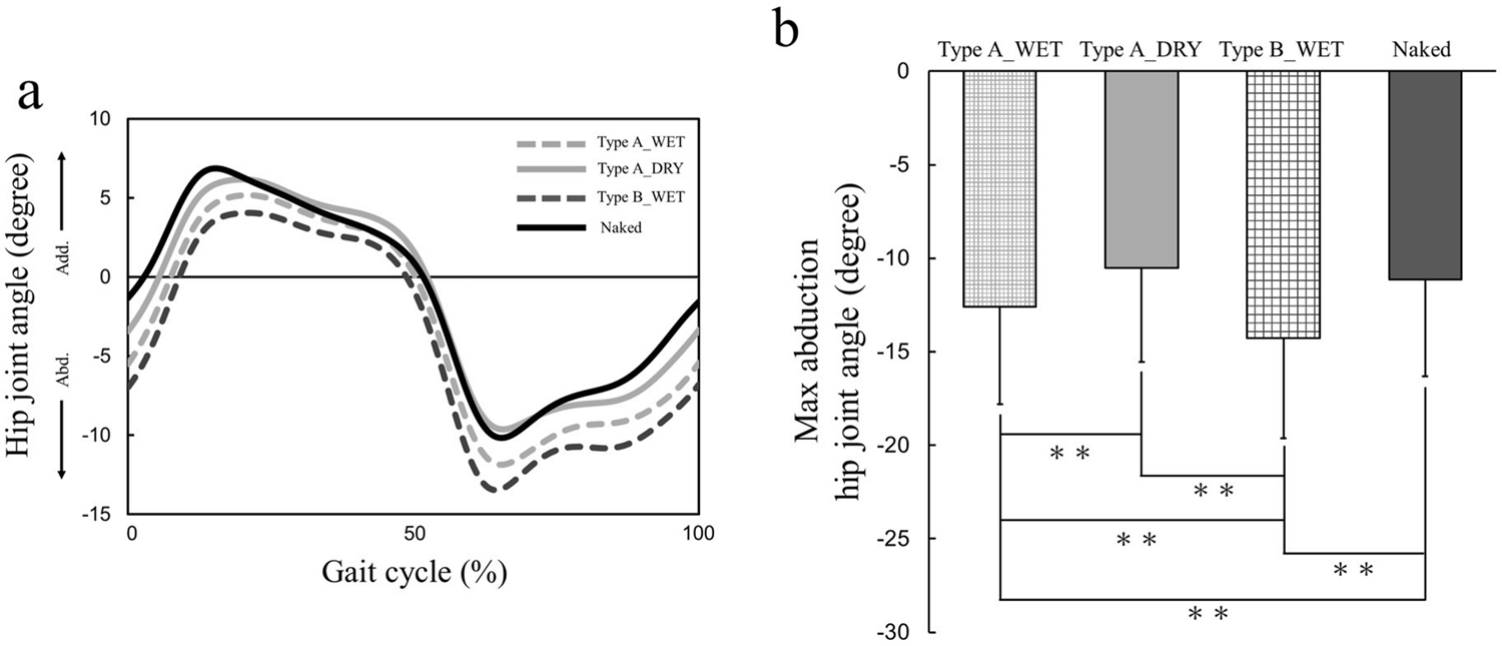
(**a**) Mean time series across subjects at each condition of the hip joint angle in the frontal plane during a gait cycle. *Abd* abduction, *Add* adduction. (**b**) Mean and standard deviation of the mean of the normalized maximum abduction hip joint angle for the four dress conditions. *p < 0.05; **p < 0.01.

Figure 2a shows the result of the average hip-joint angle in the frontal plane (abduction/adduction) in a gait cycle. Wearing diapers under wet conditions results in greater abduction than wearing diapers under dry conditions and being naked (i.e., not wearing a diaper). The dress condition had a significant effect on the maximum abduction hip-joint angle in a gait cycle (*F*_(2.45, 61.33)_ = 38.82, *p* < 0.01, ES = 0.61), and a post hoc comparison revealed that the maximum abduction hip-joint angle in the Type B_WET condition was significantly greater than that in the other three conditions (*p* < 0.01) (Fig. 2b).

### Spatiotemporal parameters

**Figure 3:**
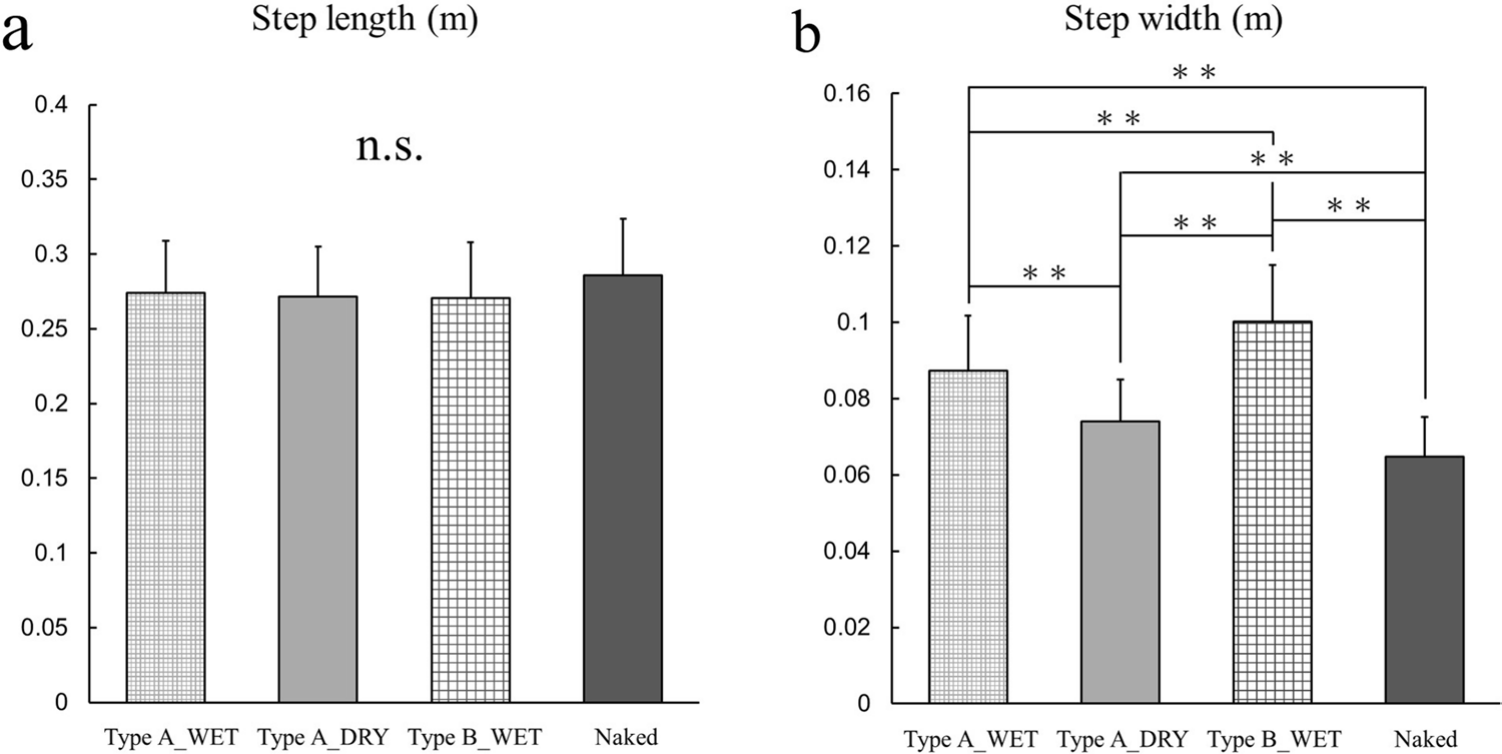
Mean and standard deviation of the mean of the normalized (**a**) step length and (**b**) step width for the four dress conditions. *p < 0.05; **p < 0.01.

Figure 3 shows the average spatiotemporal data. There were no differences in the step length under each condition (Fig. 3a). Conversely, the step width had a significant effect on the dress conditions (step width: *F*_(3, 75)_ = 114.22, *p* < 0.01, ES = 0.82), and there were significant differences among all dress conditions (Fig. 3b). Furthermore, the step width in the Type B_WET condition was significantly greater than that in the other three conditions.

## Discussion

This study was conducted to investigate the influence of wearing a disposable diaper on toddlers’ gait. The gait parameter values obtained in this study (Figs. 1, 2, 3) are highly consistent with those obtained in previous studies^10–14,19,20^, indicating a certain level of validity of the measurement and calculation of gait parameters. We hypothesized that wearing diapers had an effect on toddlers’ gait compared to not wearing diapers owing to the diaper’s high bulk stress on the lower limb, which has a greater effect on toddlers’ gait than low stress.

The pelvic angle in the horizontal plane in a gait cycle tended to increase the external rotation with the bulk stress on the lower limb (Fig. 2a). Additionally, the maximum external rotation on the pelvis was significantly greater when wearing Type B_WET than when wearing Type A_DRY, Type A_WET, or walking naked; thus, the maximum external rotation on the pelvis when walking naked was smaller than that when wearing diapers (Fig. 2b). Grigoriu et al. reported that excessive external rotation of the pelvis is a common finding in patients with cerebral palsy having an unusual mature gait^21^. Therefore, wearing wet diapers may affect the pelvis angle during gait and promote inefficient walking. Furthermore, the effect may be greater with diapers having a higher bulk stress than with diapers having a low stress because there were significant differences between Type A_WET and Type B_WET with regard to maximum external rotation on the pelvis.

The hip-joint angle in the frontal plane in a gait cycle was also found to tend to increase abduction with an increase in the bulk stress on the lower limb (Fig. 3a). The maximum abduction hip-joint angle in a gait cycle was significantly greater when wearing Type B_WET than when wearing Type A_DRY, Type A_WET, or walking naked. By contrast, walking without diapers resulted in a smaller hip abduction than walking with diapers (Fig. 3b). In other words, wearing diapers having a high bulk stress may result in greater hip abduction than wearing diapers having a low stress. In the toddlers’ gait, the hip-joint angle in the frontal plane changed with walking experience, and abduction decreased with increased walking experience^22,23^. Consequently, wearing wet diapers may have adverse effects on the development of a mature gait.

There were no differences in step length with the dress condition. Conversely, there were significant differences in step width among all dress conditions. The step width in the naked condition was the narrowest compared to that when wearing Type A_DRY, Type A_WET, and Type B_WET diapers (in order). These results are consistent with those reported in previous studies^18,24^. It follows that the effect of wearing diapers on gait is greater for step width than for step length; thus, the step width may be suitable for evaluating the gait in toddlers wearing diapers. The step width generally tends to be narrow with increasing walking experience, as in mature gait. However, the step width when wearing diapers under the dry or wet condition was much wider than that when not wearing diapers. Therefore, wearing diapers may result in inefficient walking in toddlers. Moreover, the effect may result in a wider step width when wearing diapers that have a higher bulk stress than when wearing diapers with low stress because there were significant differences between Type A_WET and Type B_WET.

The relationship between wearing diapers and gait parameters in toddlers based on the aforementioned results suggests that wearing diapers may lead to unstable and inefficient gait patterns. This is consistent with previous studies reporting that wearing diapers has an adverse effect on the development of lower limb movements in infants^17,18,24^. In addition, our study is the first to reveal the joint dynamics when wearing diapers and the effect of different bulk stresses on lower limb gait in toddlers. The present results suggest that reducing the magnitude of stress is important for toddlers’ gait because a diaper with a high bulk stress has a greater influence on joint dynamics and step width than one with a low stress. Thus, even with these limited results, our findings should contribute toward better diaper designs for toddlers that do not hinder the natural movements of the lower limb during gait. As the diaper design can influence such movements, optimizing the clothing design may contribute to the proper development of the movements of infants.

This study had several limitations. First, it did not evaluate how the interaction between the shape and the bulk of diapers affects the development of movement; thus, a future study must use different types of diapers to identify their relationship. Second, we should consider whether the compressive load for bulk is the ideal assessment for the lower limb in gait. In this study, although we used the physical property of diapers measured in a mechanical test as the influence of lower limb movement in gait, we did not directly measure the bulk stress of diapers when the toddlers were walking and the extent of the effect on the lower limb is still unclear. Therefore, we should prove the direct effect of diapers for lower limb in gait using methods such as numerical simulation of the human gait model in a further study. Third, this study did not fully observe sex differences in gait parameters because of the relatively small sample size (N = 26). Although this study was well balanced in terms of the sex of the subjects (male:female = 13:13), a future study with a larger sample size is required to perform a satisfactory statistical analysis of the effect of sex differences on gait parameters. In addition, it will be beneficial to assess the movement of not only the lower extremity but also the whole body, including the upper extremity, to synthetically evaluate gait in toddlers.

In conclusion, the present study demonstrated that the walk of a toddler when wearing a diaper differs from that when naked owing to the effect of the bulk of the diaper on the lower limb. Moreover, a high bulk stress has a greater influence than that of a low bulk stress on joint dynamics and step width. Therefore, our findings suggest that wearing diapers with high bulk stress may inhibit the natural gait patterns of toddlers.

## Methods

### Participants

Table 2 lists the characteristics of the participating toddlers. Twenty-six healthy toddlers (13 boys and 13 girls, age: 19.2 ± 0.9 months) participated in this study. All toddlers were independent walkers; the average walking experience was 6.8 ± 1.6 months. The exclusion criteria were as follows: does not usually wear disposable diapers, birth weight less than 2.5 kg, or severe illness during the first year of life. This study was carried out in accordance with the guidelines proposed in the Declaration of Helsinki, and the study protocol was approved by the Human Research Ethics Committee, Kao Corporation (Research Number T226-190615). The parents provided written informed consent by reading and signing a consent form.

**Table 2:** Characteristics of participating toddlers. Data are shown as mean ± SD.

	All (N = 26)	Male (N = 13)	Female (N = 13)
Age (months)	19.2 ± 3.6	18.6 ± 4.2	20.0 ± 4.2
Height (cm)	80.3 ± 5.0	79.7 ± 5.7	81.0 ± 4.5
Weight (kg)	11.0 ± 1.3	10.6 ± 1.5	11.3 ± 5.0
Walking experience (months)	6.8 ± 1.5	6.5 ± 1.7	7.1 ± 1.3

### Data collection

**Figure 4:**
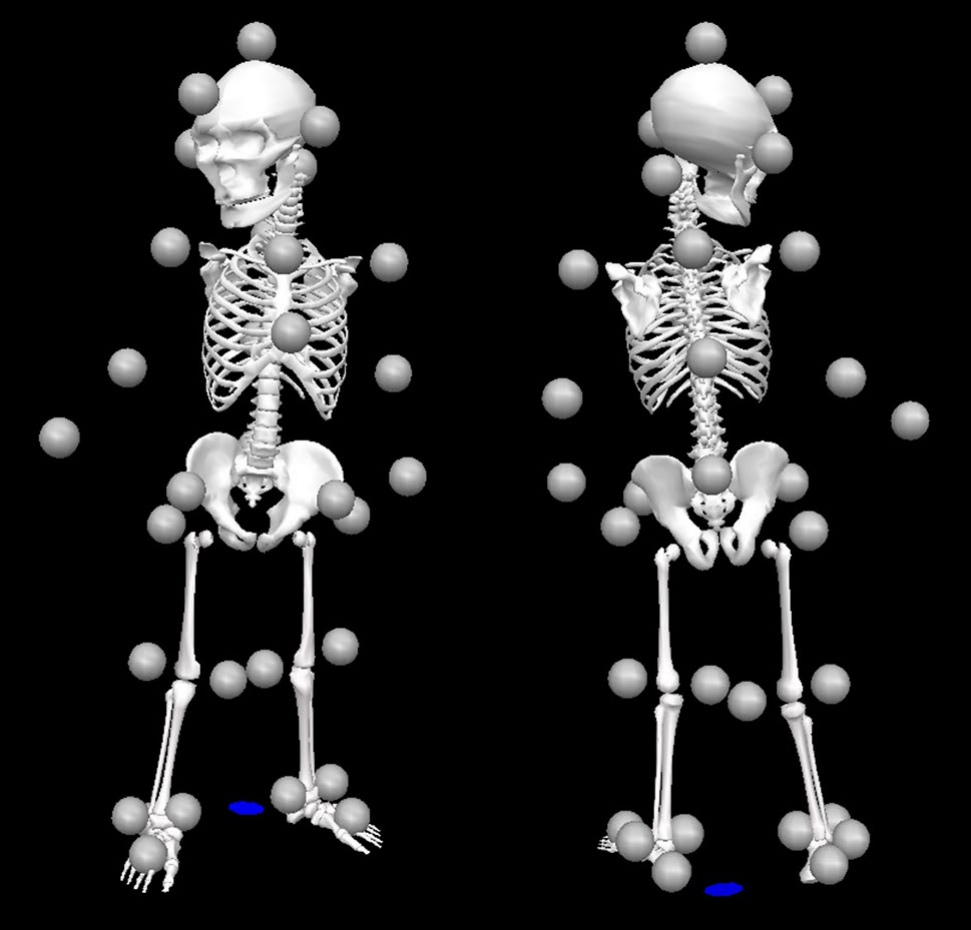
Nine-segmented body model constructed from 32 markers.

Data were collected at the School of Design, Shanghai Jiao Tong University (Shanghai, China), using a Vicon motion system. A walkway (2 × 5 m) was surrounded with 12 automated infrared reflective cameras (Vicon Vantage V5, 100 Hz). Three-dimensional (3D) positional data were obtained using reflective markers and a 3D motion capture system. A total of 32 infrared reflective markers were attached in accordance with the guidelines of the Visual 3D software (C-Motion Inc., Rockville, MD, USA). The 9-segmented body model (head, trunk, pelvis, thighs, shanks, feet) was based on the standard Visual 3D model but adapted to match the posture of toddlers (Fig. 4). The toddlers walked barefoot at self-selected timings on a walkway. Four randomized conditions were tested: wearing an A-type diaper (Type A_DRY), wearing an A-type diaper under wet conditions (Type A_WET), wearing a B-type diaper under wet condition (Type B_WET), and wearing nothing (Naked). The disposable diaper used in testing was a common commercially available brand in L size. The diapers under wet conditions contained saline solution (160 g) just before measurements. The saline solution was used to simulate urination. The diapers were changed by parents or researchers. At least ten trials were conducted under each condition, with two caregivers standing at each end of the walkway to encourage the child to walk toward them in a straight line. Trials where the child stopped walking, turned around, ran, or fell were excluded.

### Bulk stress measurement

**Figure 5:**
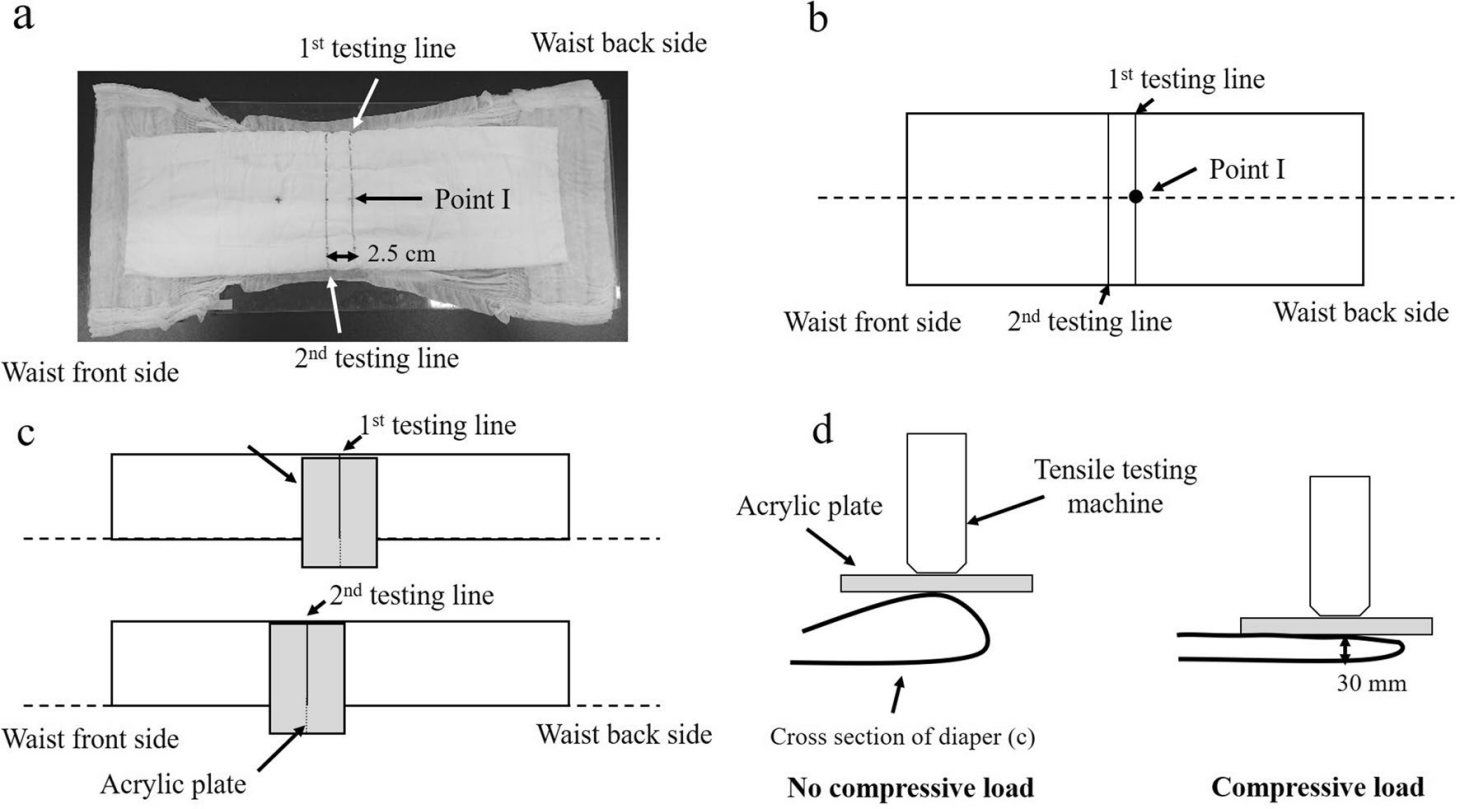
Method for measuring bulk stress. (**a**) The diaper was opened, extended fully, and marked with two straight lines. (**b**) The diaper was folded to half size along the dotted line as illustrated. (**c**) A rectangular acrylic plate was placed on the diaper. (**d**) The center of the plate was compressed vertically downward by a tensile testing machine at a speed of 100 mm/min until the thickness of the diaper decreased to 30 mm.

The bulk stress in each diaper was measured using the following steps^26^. The diaper was opened and extended. Two straight lines were marked as guides for the two testing sites, as shown in Fig. 5a. The first testing line was parallel to the shorter direction of the diaper and passed through the center position of the diaper (Point I). The second testing line was parallel to the shorter direction and was 2.5 cm away from the first line to the direction of the front side of the diaper. Figure 5b shows that the diaper was folded into half its size along the dotted line, that is, along the line parallel to the longer direction and passing through Point I. The diaper was placed on a horizontal surface so that no wrinkles or bends were formed. A rectangular acrylic plate with a width of 5 cm, length of 15 cm, and weight of 28.7 g was placed on the diaper, as shown in Fig. 5c,d: (1) the longer side of the plate was perpendicular to the direction of the diaper, (2) the longitudinal center line of the plate was adjusted on the first and second testing lines on the diaper, and (3) the longitudinal center of the plate was adjusted to the highest point of the diaper, as shown in Fig. 5d.

The center (both longer and shorter directions) of the plate was compressed vertically by a tensile testing machine (Autograph AGS-X, Shimadzu Corporation) at a speed of 100 mm/min until the thickness of the diaper was 30 mm. The compressive load was measured and recorded when the thickness of the diaper was 30 mm (Fig. 5d). The average compressive load measurements on the first and second testing lines were used as the compressive load for the bulk. The higher the value, the higher was the stress on the lower limbs of the toddlers. Table 2 lists the specifications of each diaper.

### Data analysis

**Figure 6:**
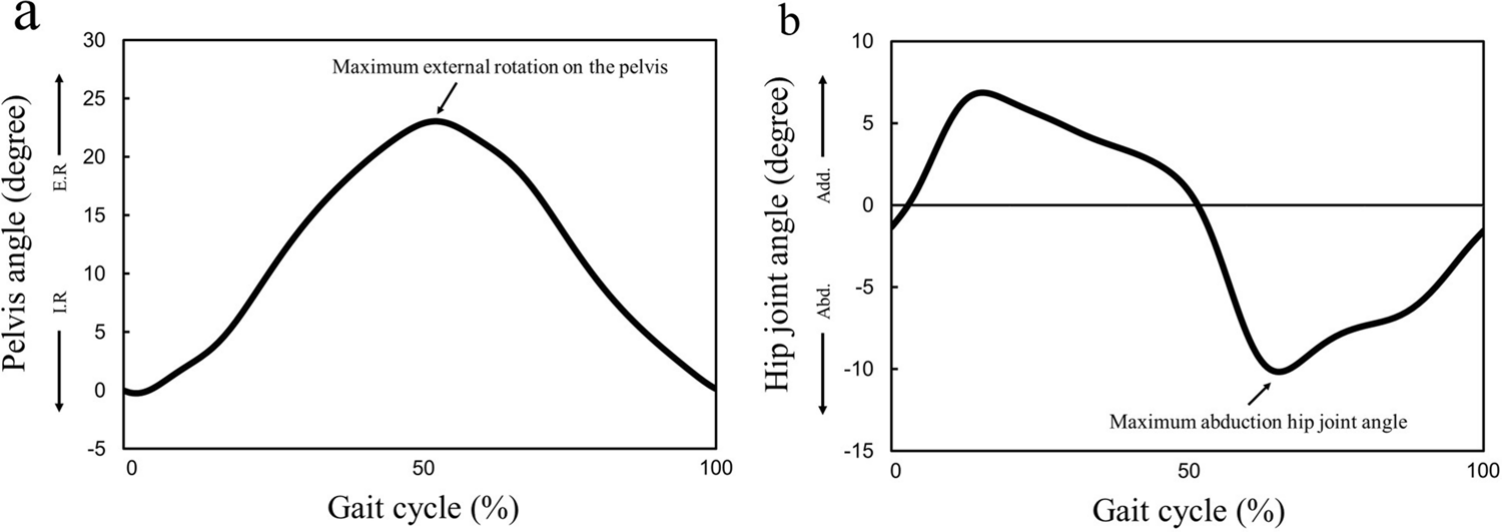
Illustration of kinematics parameters: (**a**) maximum external rotation on the pelvis in a gait cycle and (**b**) maximum abduction hip joint angle in a gait cycle.

From each successful trial, more than five complete gait cycles per condition were retained for analysis without distinguishing between the left and the right gait cycles. The raw data were digitally filtered using a fourth-order Butterworth filter with zero lag and cut-off frequencies of 10 Hz for the positional data. The pelvic angle on the z-axis (e.g., internal–external rotation) and hip-joint angles on the y-axis (e.g., abduction–adduction) during one gait cycle were calculated using a Cardan sequence of rotations (X–Y–Z) from the trajectories measured in each trial. The angles were time-normalized by the gait cycle duration determined from the trajectories of the heel and toe markers and divided into 101 variables ranging from 0 to 100%. We considered the maximum external pelvic rotation and maximum hip-joint abduction angle in a gait cycle for assessing the toddlers’ gait in the lower limbs, because the timing of their appearance in a gait cycle is most affected by the bulk stress of the diapers when the toddlers’ legs come in contact with the diapers (Fig. 6).

The step length and step width were determined to help understand the gait characteristics. Low-pass filtering and calculation of the variables were performed using the Visual 3D software. The obtained kinematics and spatiotemporal parameters were averaged for each subject.

### Statistics

A one-way repeated-measure analysis of variance (ANOVA) was used to assess the effect of the four dress conditions (Type A_WET, Type A_DRY, Type B_WET, and Naked) for each index. To assess the assumptions of variance, Mauchly’s test of sphericity was performed using all ANOVA results. A Greenhouse–Geisser correction was performed to adjust the degrees of freedom if an assumption was violated, and a Bonferroni post hoc multiple comparison test was performed to ascertain if a significant effect occurred. Statistical significance was set at *p* < 0.05. SPSS for Windows version 22 (IBM, Armonk, NY, USA) was used for all statistical analyses.

## Data Availability

Participants of this study did not agree for their data to be shared publicly, so supporting data is not available.
